# Braconinae parasitoids (Hymenoptera, Braconidae) emerged from larvae of *Lobesia botrana* (Denis & Schiffermüller) (Lepidoptera, Tortricidae) feeding on *Daphne gnidium* L.

**DOI:** 10.3897/zookeys.587.8478

**Published:** 2016-05-10

**Authors:** Augusto Loni, Konstantin G. Samartsev, Pier Luigi Scaramozzino, Sergey A. Belokobylskij, Andrea Lucchi

**Affiliations:** 1 Department of Agriculture, Food and Environment, Pisa University, Via del Borghetto, 80-56124 Pisa, Italy; 2 Zoological Institute, Russian Academy of Sciences, Universitetskaya nab., 1, St Petersburg 199034, Russia; 3 Museum and Institute of Zoology, Polish Academy of Sciences, Wilcza 64, Warszawa 00–679, Poland

**Keywords:** European grapevine moth, idiobiont ectoparasitoids, Italy, natural environment, spurge flax, Thymelaeaceae, Tuscany

## Abstract

*Bracon admotus* Papp, 2000, and three species of the genus *Habrobracon* Ashmead, 1895, *Habrobracon concolorans* (Marshall, 1900), *Habrobracon hebetor* (Say, 1836) and *Habrobracon pillerianae* Fischer, 1980, were obtained from the larvae of *Lobesia botrana* (Denis & Schiffermüller, 1775) (Lepidoptera, Tortricidae) feeding on *Daphne gnidium* Linnaeus, 1753 (Thymelaeaceae) in the natural reserve of Migliarino-San Rossore-Massaciuccoli (Pisa-Central Italy). *Bracon admotus*, *Habrobracon concolorans* and *Habrobracon pillerianae* were found for the first time to be associated with *Lobesia botrana*, while *Habrobracon hebetor* was reared for the first time from the larvae of *Cryptoblabes gnidiella* (Millière, 1867) (Lepidoptera, Pyralidae, Phycitinae) that was found on the same host plant. *Bracon admotus* and *Habrobracon pillerianae* are new to the fauna of Italy and Western Europe. A key is proposed for the determination of *Habrobracon* species reared from *Lobesia botrana* and related Palaearctic species of this genus. *Habrobracon lineatellae* Fisher, 1968 is considered as a valid species.

## Introduction

With about 2,500 species from almost two hundred genera, Braconinae is one of the largest subfamilies within the family Braconidae (Shaw and Huddleston 1991, Quicke 2015). This parasitoid group has a worldwide distribution with the largest number of taxa found in the Old World tropics (Quicke 2015). Most Braconinae species are idiobiont ectoparasitoids that develop on concealed or semi-concealed hosts mainly represented by late larval instars of numerous Coleoptera and Lepidoptera taxa, and more rarely by sawflies (Hymenoptera, Symphyta) and flies (Diptera), predominantly gall midges (Cecidomyiidae) and fruit flies (Tephritidae). Females of Braconinae are synovigenic, practicing additional host feeding and laying large eggs. The larvae develop as gregarious or solitary parasitoids (Shaw and Huddleston 1991, Quicke 2015). Their host range includes many species with a large variety of habits, but all the victims show a certain degree of concealment in the tissues of annual and biennial plants, including galls, rolled leaves, inflorescences, seeds, stems and, more rarely, leaf mines or hard wood tissues (Quicke 2015). Several species of Braconinae attack pests of an economic interest, such as stored products and field crops pests (Quicke 2015).

Among Braconinae, *Bracon* Fabricius, 1804 is a cosmopolitan and very common genus composed of the largest number of species. European fauna includes about 200 species of *Bracon* living on larvae of Lepidoptera, Coleoptera and Diptera (van Achterberg 2013). This genus is considered as a para- or also a polyphyletic group, predominantly of small and middle-sized species (Quicke 2015) spread over several subgenera. *Habrobracon* Ashmead, 1895 was once considered to be a distinct genus (Quicke 2015, Papp 2008, Yu et al. 2012) or one of the *Bracon* subgenera (Shenefelt 1978, Tobias 1986, Belokobylskij et al. 2012, Broad et al. 2012, Ameri et al. 2013, 2015, Zargar et al. 2015) or sometimes is considered as a synonym (Marsh 1979, van Achterberg 2013).

In agreement with the latest opinions of Papp (2008) and Quicke (1987, 2015) recorded in the World Catalogue by Yu et al. (2012), we consider *Habrobracon* as a separate genus in spite of the intermediate position of *Bracon variegator* Spinola and various Nearctic species between the two genera together with the lack of diagnostic characters at the generic level.

*Habrobracon* is a worldwide group of small to very small wasps (Quicke 1987). Twenty-one *Habrobracon* species have been recorded in the Western Palaearctic (Papp 2008), some of which have a host range of more than 50 host-species with a high ecological flexibility (Tobias 1986, Yu et al. 2012, Beyarslan et al. 2014). *Bracon admotus* Papp, 2000 was originally included in the *Bracon obscurator* species-group within the subgenus *Glabrobracon* Fahringer, 1927.

This paper presents the results of a study carried out in the natural reserve of Migliarino-San Rossore-Massaciuccoli in the province of Pisa (Tuscany, Italy). All over this area there is a large population of spurge flax, *Daphne gnidium* Linnaeus, 1753, a small shrub of the family Thymelaeaceae, whose sprouts, flowers and infructescences host a large community of moth larvae, most of which are represented by *Lobesia botrana* (Denis & Schiffermüller, 1775) (Lepidoptera: Tortricidae). This species which was recently defined as European grapevine moth (EGVM) is a major pest of grapes in the Mediterranean basin and had recently been found in the Americas (Ioriatti et al. 2011, 2012).

Predators and parasitoids associated with this moth have been studied in various European countries, and more than a hundred works have been published on the subject. To date there is still an incomplete list of the natural enemies of *Lobesia botrana*, and information about these enemies is contained in some works published in the twentieth century, when the moth caused the first extensive damage to European vineyards (Marchal 1912, Voukassovitch 1924, Leonardi 1925, Boselli 1928, Stellwaag 1928, Thompson 1946, Coscollà 1997, Hoffmann and Michl 2003, Villemant et al. 2011).

In Italy, about thirty studies have been published reporting on 89 species of parasitoids living on *Lobesia botrana* (Del Guercio 1899, Catoni 1914, Silvestri 1912, Nuzzaci and Triggiani 1982, Delrio et al. 1987, Luciano et al. 1988, Pinna et al. 1989, Lozzia and Rigamonti 1991, Roat and Forti 1994, Marchesini and Dalla Montà 1994, Colombera et al. 2001, Bagnoli and Lucchi 2006, Lucchi and Santini 2011).

One of the problems with identifying the parasitoid complex of a pest is that the much of the data regards pests in agroecosystems, which usually simply represent pests outside of their natural environment. This is also valid for the European grapevine moth. The hundred or so published papers on its parasitoids only concern the dynamics in the vineyard. There are only three exceptions, all by Italian authors (Nuzzaci and Triggiani 1982, Luciano et al. 1988, Lucchi and Santini 2011) who have tried to highlight the population dynamics of the moth and its parasitoids on *Daphne gnidium*, which is its typical spontaneous host plant in wild habitats.

This paper reports on a study carried out in 2014 and 2015. It provides new information on one species of *Bracon* and three species of *Habrobracon* obtained from the larvae of *Lobesia botrana* living on *Daphne gnidium* in a natural reserve in Tuscany (Italy). The massive presence of this important pest in a wild area offers a very interesting environmental context where to perform observations on the relationships among this phytophagous and its parasitoids complex in comparison with the cultivated field.

## Methods

### The environmental context

Weekly surveys were carried out in 2014 and 2015 in the natural reserve of Migliarino-San Rossore-Massaciuccoli, which covers around 23,000 hectares in the provinces of Pisa and Lucca (Tuscany, Italy) (http://www.parcosanrossore.org/). The landscape has a variety of environments, such as sandy shores and dunes stretching for about 23 km along the coast, wetlands with marshes, rivers, lakes, ponds, and forests. The Tyrrhenian Sea delimits the western border, the Lake of Massaciuccoli, the northern border, and the river Arno, the southern border. To the east, the landscape changes gradually from wild to rural areas. The distance from the east border to the west coast varies ranging from 6 to 10 kilometers.

In the back dunes and the thermophile Mediterranean wood, dominated by pine tree and holm oak, *Daphne gnidium* L. (Malvales, Thymelaeaceae) shrubs are widespread, covering the spaces where the sun can easily penetrate and the soil is mostly sandy. From March to October, sprouts, flowers and infructescences, depending on the period, host a wide and diverse community of Lepidoptera, mainly represented by *Lobesia botrana*.

### Sampling methodology

**Figure 1.**
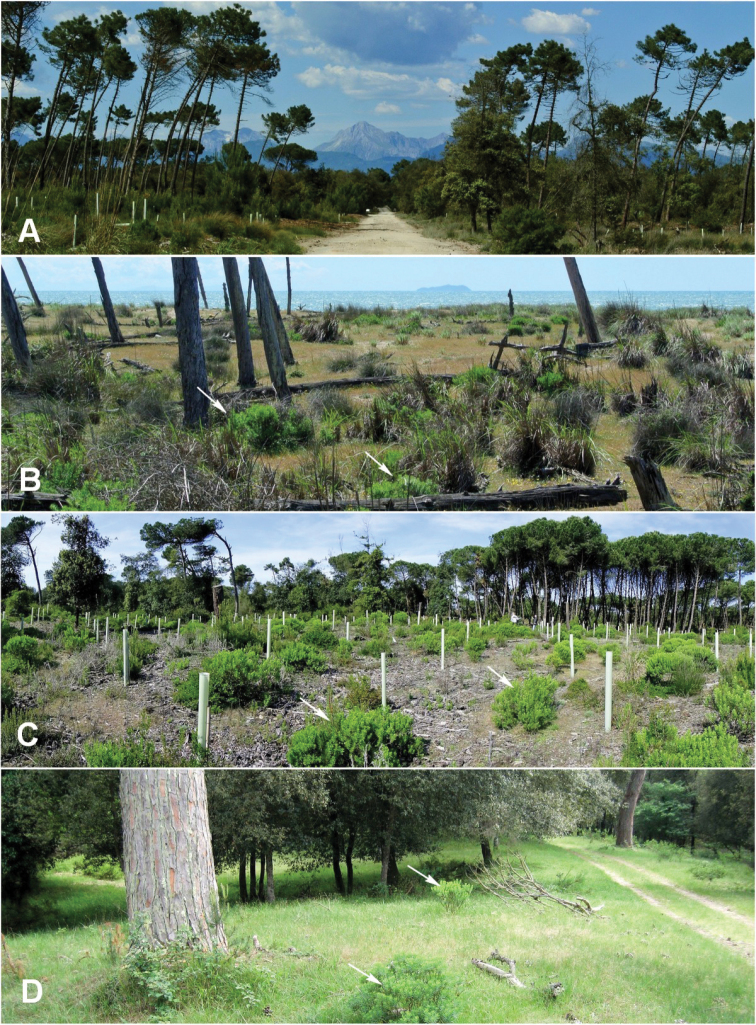
Natural Reserve of San Rossore (Pisa): different habitats of the experimental area. **A** Landscape **B** Fore dune close to sandy shores **C** vegetation of established dunes **D** Wooded area with holm oak. White arrows indicate plants of *Dapne gnidium*.

**Figure 2.**
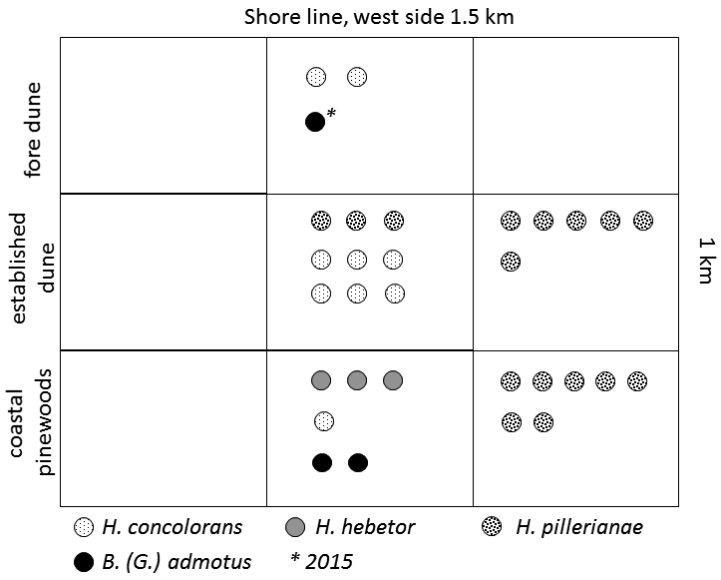
Sampling grid, with distribution of specimens collected.

**Figure 3.**
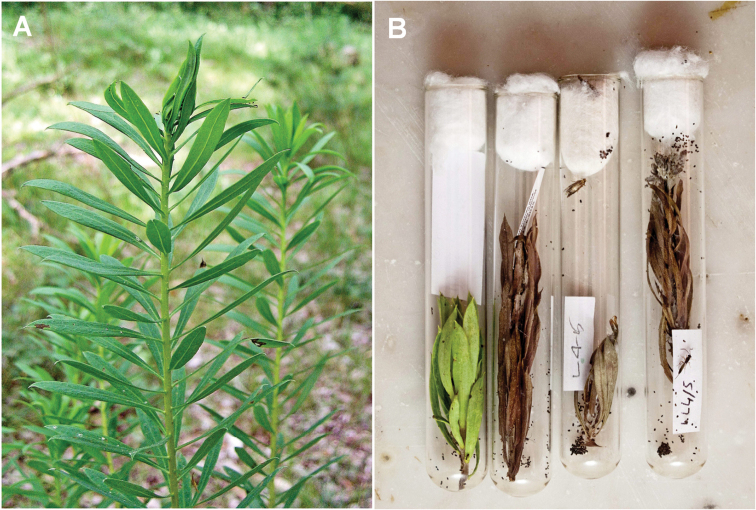
Sprouts of *Daphne gnidium* infested by moth larvae. **A** Field situation **B** Nests stored in vials until emergence of the moth or the parasitoid.

An experimental area was selected, delimited by the following four geographical points 43.733642 N, 10.277524 E; 43.712864 N, 10.279648 E; 43.732913 N, 10.292371 E; 43.720101 N, 10.293094 E (DDM) and characterized by various habitats (Fig. 1). We subdivided this area in a hypothetical grid of 3 × 3 rectangles, each one being 500m x 300m, thus the sampling was replicated in each habitat typology three times (Fig. 2). In each of the nine rectangular sites, transects of 200 meters in length were organized along where *Daphne gnidium* sprout sampling was carried out. On a weekly basis in each transect 20 infested sprouts were sampled from ten plants (two per plant) (Fig. 3A). In 2014, sampling was begun on May 22 and concluded on October 23. In 2015, surveys started on May 14 and finished on October 23. On the same or the following day of the sample collection, the sprouts were analyzed under a stereomicroscope to isolate the preimaginal stages of the moths. Then each individual specimen was stored inside a glass vial, recording its precise developmental stage. Until the moth or the parasitoids emerged, samples were stored in boxes and maintained in an unheated room with an open window, to maintain as similar environmental conditions as possible to those of the sampling sites (Fig. 3B).

Among the huge number of parasitoid species that emerged during the rearing period, we focused on a small group of individuals belonging to the genera *Bracon* and *Habrobracon*, examining their role in the observed context, and their geographical distribution. The species mentioned in this work were identified by K. Samartsev. The specimens are now stored both at the Laboratory of Insect Taxonomy of the Zoological Institute of the Russian Academy of Sciences (St Petersburg, Russia) and at the Department of Agriculture, Food and Environment of Pisa University (Italy). For a description of the morphological features of the species, we referred to Quicke (2015). The following abbreviations are used in the paper:

POL postocellar line


OOL ocular-ocellar line


Od maximum diameter of lateral ocellus


Wing venation nomenclature follows van Achterberg (1993). Those terms that follow Tobias’s nomenclature (1986) are:

1-R1 metacarp


2-SR first radiomedial vein


2-SR+M second medial abscissa


3-SR second radial abscissa


m-cu recurrent vein


r first radial abscissa


SR1 third radial abscissa


marginal cell radial cell


## Results

In 2014 and 2015 approximately 4,200 infested sprouts of *Daphne gnidium* were examined, obtaining 1,254 larvae of *Lobesia botrana* in 2014, and 942 in 2015. In 2014 30 specimens of two genera were obtained, *Bracon* spp. and *Habrobracon* spp., emerging from the larvae of *Lobesia botrana* and *Cryptoblabes gnidiella* (Millière, 1867) (Lepidoptera: Pyralidae), one of the other moths found on *Daphne gnidium*, while in 2015 we obtained only one specimen of *Bracon*.

These specimens represent approximately 11% of the parasitoid complex emerging from all samples in 2014, the majority of which were Ichneumonidae. They were mainly represented by the species *Campoplex capitator* Aubert, 1960, occurring across all sites and which contributed for more than 58% of the total number of parasitoids found in 2014 (Table 1) and more than 73% in 2015. In 2014 we obtained 2 males of *Bracon admotus* Papp, 7 females and 2 males of *Habrobracon concolorans* (Marshall, 1900), 3 females of *Habrobracon hebetor* (Say, 1836), 9 females and 7 males of *Habrobracon pillerianae* Fischer, 1980. Each species of *Habrobracon* was distributed at most over three of the nine collecting sites, the two specimens of *Bracon admotus* were collected only in one site (Fig. 2). In 2015 we obtained one male of *Bracon admotus*.

**Table 1.** List of the main parasitoids emerged from the *Daphne gnidium* sprouts (2014).

Campoplex capitator Auber, 1960 (Ichneumonidae)	Other Ichneumonidae	Bracon spp. Habrobracon spp. (Braconinae)	Other Braconidae (Cheloninae and Rogadinae)	Chalcidoidea	Diptera Tachinidae
126	35	30	9	13	7

### Bracon (Glabrobracon) admotus

Taxon classificationAnimaliaHymenopteraBraconidae

Papp, 2000

Bracon admotus Papp, 2000: 237; Yu et al. 2012.

#### Material examined.

2 males, October 14, 2014; 1 male, October 1, 2015.

This is the first record for Italy and Western Europe and *Lobesia botrana* represents a new host for this parasitoid. The most important characters for distinguishing of *Bracon admotus* from the similar species of *Bracon variator* and *Bracon obscurator* species groups are: longitudinal diameter of eye 3.3–3.4 times (about 4.4 times in males) longer than malar space (front view); hypoclypeal depression 1.5–1.6 times (1.6–1.7 times in male) as wide as distance from depression to eye; mesosoma short, about 1.4 times (about 1.5 times in males) longer than maximum height; face and frons evenly granulate; vein r issued clearly before middle of pterostigma; first metasomal tergite (if measured from basomedian tubercle) 1.1–1.3 times as long medially as its apical width; furrow of first tergite and suture between second and third tergites crenulate; metasoma usually completely smooth.

Figure 5 reports various morphological details of the species.

### Habrobracon concolorans

Taxon classificationAnimaliaHymenopteraBraconidae

(Marshall, 1900)

Bracon concolor Thomson, 1892: 1807; Yu et al. 2012.
Bracon concolorans Marshall, 1900: 345 (new name for Bracon concolor Thomson, 1892 nec Bracon concolor Walker, 1871); Yu et al. 2012.
Habrobracon nigricans Szépligeti, 1901: 181; Yu et al. 2012.
Habrobracon mongolicus Telenga, 1936: 130, 342; Yu et al. 2012.

#### Material examined.

1 female, May 29, 2014; 4 females from the same host larva, July 4, 2014; 2 males from the same host larva, July 23, 2014; 2 females, October 2, 2014.

*Habrobracon concolorans* is a Trans-Eurasian species (Samartsev and Belokobylskij 2013), widely distributed in the Palaearctic region and has been recorded in the following countries (Yu et al. 2012): Europe: Ireland, United Kingdom, Sweden, Denmark, France, Spain, Italy, including Sicily (Zappala et al. 2012b), Lithuania, Russia (Kaliningrad and Astrakhan provinces), Poland, Czech Republic, Slovakia, Hungary, Romania, Moldova, Bulgaria, Croatia, Greece; Middle East: Turkey, Cyprus, Jordan (Al-Jboory et al. 2012; Zappalà et al. 2013), Iran; Caucasus: Russia (Ciscaucasia: Krasnodar Territory), Georgia, Armenia, Azerbaijan; Central Asia: Kazakhstan, Turkmenistan (Papp 2008), Kyrgyzstan, Mongolia (Papp 2009); Russian Far East: Sakhalin, Khabarovsk Territory, Primorskiy Territory; China: Shanxi, Ningxia, Fujian; Africa: Egypt (Zappalà et al. 2013), Tunisia (Papp 2014), Sudan (Ghoneim 2014, Mahmoud 2013).

**Figure 4.**
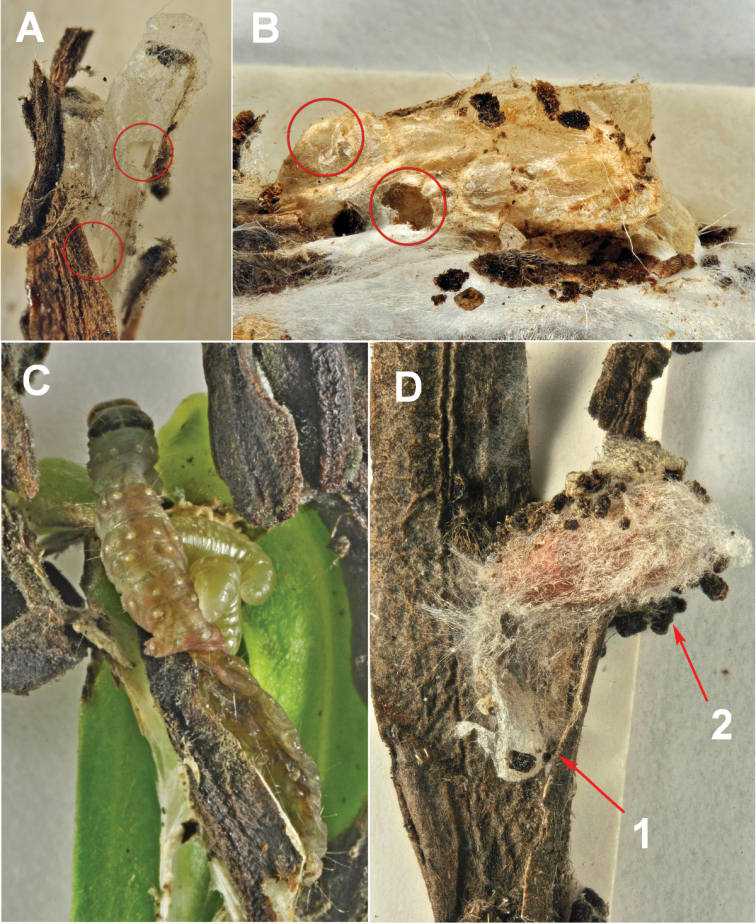
*Habrobracon* preimaginal stages on the hosts *Lobesia botrana* (**A, B, C**) and *Cryptoblabes gnidiella* (**D**). **A** *Habrobracon pillerianae* cocoons, the circles surround the exit holes **B** *Habrobracon concolorans* cocoons **C** *Habrobracon pillerianae* larvae feeding on mature larvae of *Lobesia botrana* **D** *Habrobracon hebetor* (1) and *Cryptoblabes gnidiella* (2) cocoons.

The collecting period of the *Lobesia botrana* larvae hosting *Habrobracon concolorans* ranged from May 29 to October 2. *Lobesia botrana* is considered a new host for *Habrobracon concolorans*. Also on *Lobesia botrana*, *Habrobracon concolorans* develops as ectoparasitoids of mature larvae showing both solitary and gregarious habit, with up to four individuals feeding on the same host larva (Fig. 4B). Figure 6 reports the morphological details of the species.

**Figure 5.**
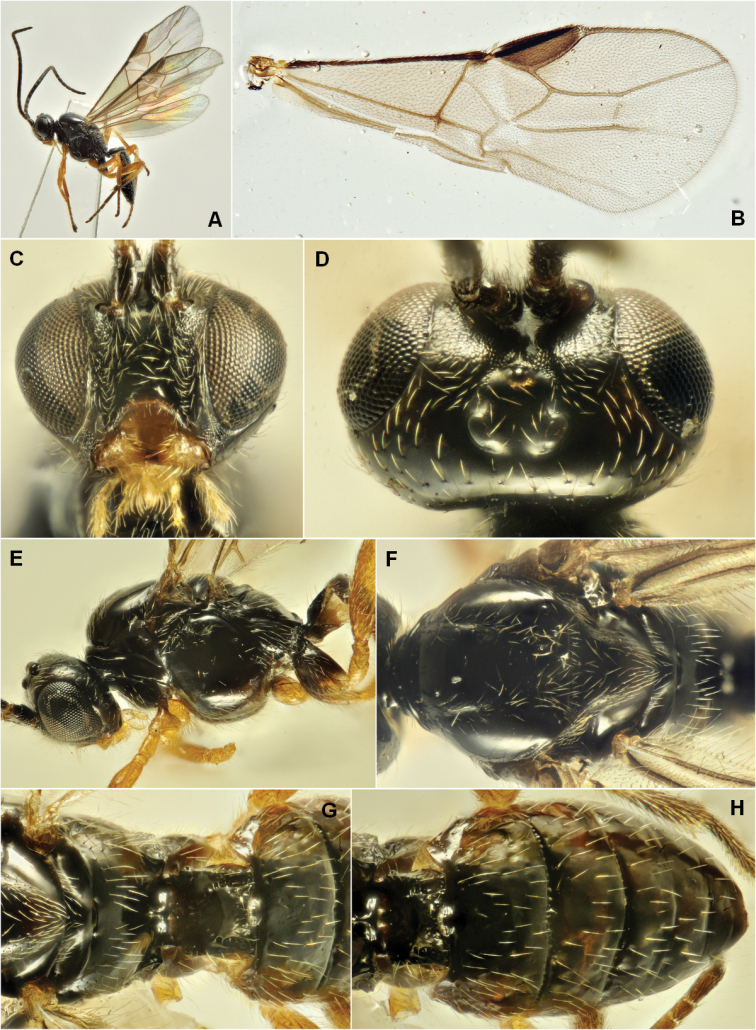
Bracon (Glabrobracon) admotus Papp 2000, male. **A** Body, lateral view **B** Fore wing **C** Head, front view **D** Head, dorsal view **E** Head and mesosoma, lateral view **F** Mesosoma, dorsal view **G** Metanotum, propodeum and basal segments of metasoma, dorsal view **H** Metasoma, dorsal view.

**Figure 6.**
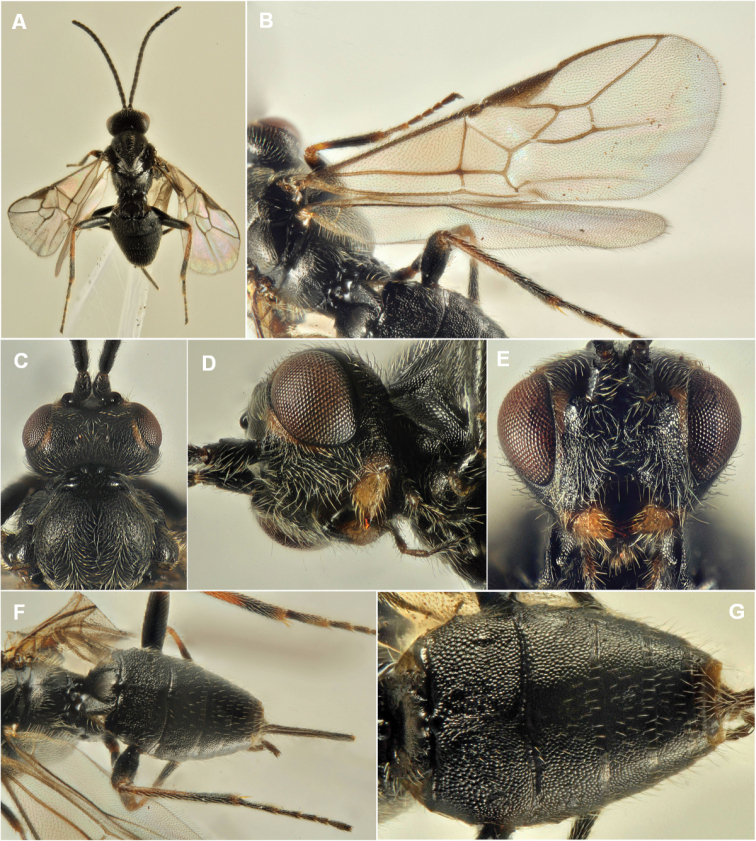
*Habrobracon concolorans* (Marshall, 1900), female. **A** Body, dorsal view **B** Wings **C** Head and mesonotum, dorsal view **D** Head, sub-lateral view **E** Head, front view **F** Propodeum and metasoma, dorsal view **G** Metasoma, dorsal view.

### Habrobracon hebetor

Taxon classificationAnimaliaHymenopteraBraconidae

(Say, 1836)

Bracon hebetor Say, 1836: 252; Yu et al. 2012.
Bracon brevicornis Wesmael, 1838: 23; Yu et al. 2012.
Bracon juglandis Ashmead, 1889: 621; Yu et al. 2012.
Habrobracon hebetor Other less valuable synonyms are listed in Yu et al. 2012.

#### Material examined.

1 female from larva of *Cryptoblabes gnidiella*, May 29, 2014; 2 females from larvae of *Lobesia botrana*, June 6, 2014.

We found two specimens on mature larvae of *Lobesia botrana*, but we also obtained one specimen by *Cryptoblabes gnidiella* (Fig. 4D), thus confirming its suitability in many environmental situations, where it can occupy a large variety of ecological niches.

**Figure 7.**
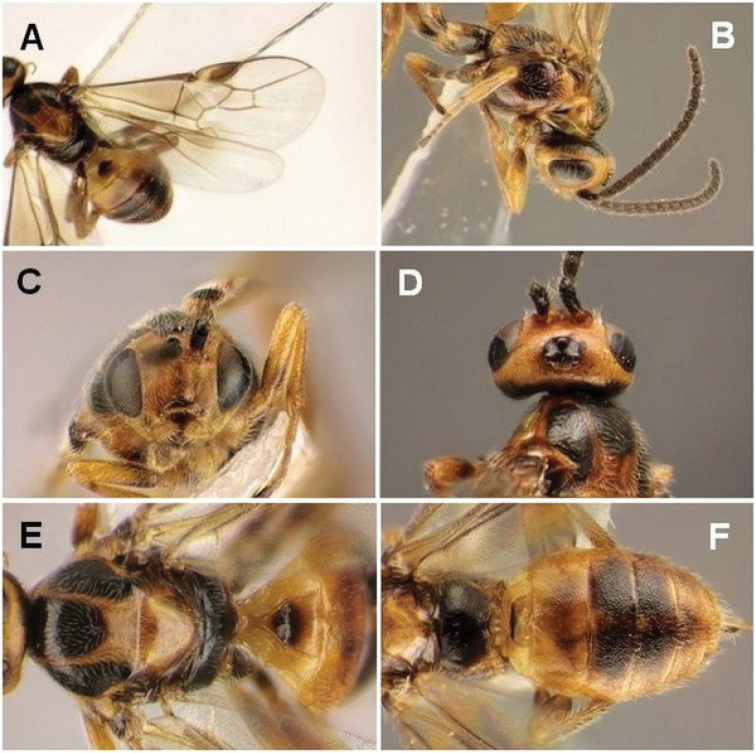
*Habrobracon hebetor* Say, 1936, female, **A** Wings **B** Head and mesosoma, lateral view **C** Head, front view **D** Head, dorsal view **E** Mesosoma, dorsal view **F** Metasoma, dorsal view.

Figure 7 shows various morphological details of this species.

### Habrobracon pillerianae

Taxon classificationAnimaliaHymenopteraBraconidae

Fischer, 1980

Habrobracon pillerianae Fischer, 1980: 150; Yu et al. 2012.

#### Material examined.

5 females and 2 males, June 27, 2014 (3 females and 1 female + 2 males from the same host larva); 2 females and 1 male (1 male and 1 female reared from the same host larva) July 23, 2014; 1 female, 4 males, July 31, 2014; 1 female September 10, 2014.

**Figure 8.**
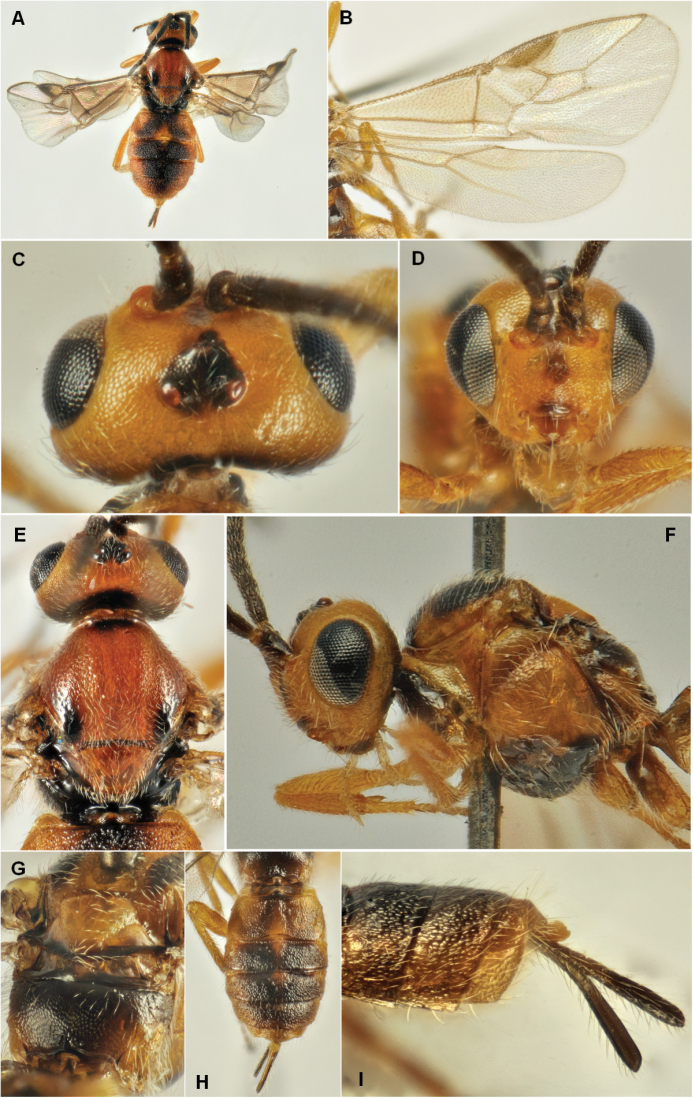
*Habrobracon pillerianae* Fischer, 1980, female. **A** Body, dorsal view, **B** Wings **C** Head, dorsal view **D** Head, front view **E** Mesosoma, dorsal view **F** Head and mesosoma, lateral view **G** Metanotum and propodeum, subdorsal view **H** Metasoma, dorsal view **I** Apex of metasoma, lateral view.

Six specimens of this species were described by Fischer (1980) which were reared from larvae of *Sparganothis pilleriana* (Denis & Schiffermüller, 1775) (Lepidoptera Tortricidae) in Ankara Province, Central Anatolia, Turkey (Fischer 1980). To date, this is the only paper reporting original information on this species (Yu et al. 2012). We obtained this species in our rearing programme from June 27 to September 10, 2014. The dates of the emergence of the specimens well fitted with those reported by Fisher, who described *Habrobracon pillerianae* from specimens collected on July 20, 1976. Also in this species the larvae developed both solitary and gregariously, with up to three individuals feeding on the same host larva (Fig. 4A, C). This is the first report of this species for Italy and Europe as well as the first association with *Lobesia botrana*. Figure 8 shows various morphological features of this species.

### Key to the *Habrobracon* species reared from *Lobesia botrana* on *Daphne gnidium* with respect to related species recorded in the Western Palaearctic

**Figure 9.**
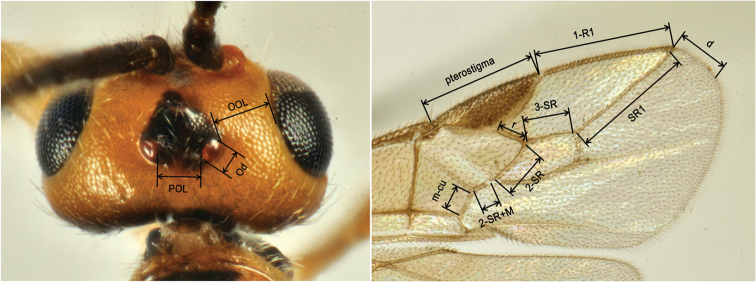
Measures adopted in the key for head. **A** (Od = Ocellar diameter, OOL = Ocular - Ocellar Line, POL = Post-Ocellar Line) and distal part of fore wing **B** (d = distance from apex of marginal cell to apex of wing).

Using the most complete key for the Palaearctic species (Tobias 1986), the specimens of *Habrobracon pillerianae* are to be identified as *Habrobracon telengai* or *Habrobracon viktorovi*, the difference between *Habrobracon concolorans* and related species is also not very clear. Therefore, it seems appropriate to indicate the position of the three identified species in the whole genus by providing the following key. Suggested key does not aim to help to identify the *Habrobracon* species groups not related with *Habrobracon concolorans*, *Habrobracon hebetor* and *Habrobracon pillerianae*. Such unrelated species are distinguished in the key couplets 1 and 5. In some couplets, additional information helping the species identification is listed after a dash ( – ). Measures adopted for the head in the key are shown in Figure 9A. Wing veins are measured excluding their junctions (Fig. 9B). The most important synonyms are given in parentheses.

**Table:** 

1	Middle lobe of mesoscutum glabrous (as in Fig. 5F). Vein 3-SR 1.1–1.3 times longer than vein 2-SR	***Habrobracon variegator* (Spinola, 1808) species group** (sensu Tobias)
–	Middle lobe of mesoscutum (often evenly) setose (Figs 6C, 7E, 8E). Vein 3-SR not longer than vein 2-SR (except *Habrobracon lineatellae*; Figs 6B, 7A, 9B)	2
2	Mesoscutum (except middle lobe posteriorly and notauli lines) and most of mesopleuron completely smooth. Vein 2-SR+M 0.5–0.7 times as long as vein 2-SR, 1.1–1.4 times as long as vein m-cu (Fig. 7A; if rarely 0.8–0.9 times then vein 1-R1 not longer than pterostigma). - Metanotum smooth (Fig. 7F). Metasomal tergites with weak sculpture, shiny (Fig. 7F). First metasomal tergite with smooth furrow (Fig. 7E)	**3**
–	Mesoscutum and mesopleuron distinctly granulose or shagreened (Fig. 6C), if sometimes with smoothed areas (Fig. 8E, 8F) then vein 2-SR+M 0.3–0.5 times as long as vein 2-SR and 0.4–0.8 times as long as vein m-cu (Fig. 8B) and first metasomal tergite usually with crenulate furrow	**4**
3	Antennae not thickened; first flagellar segment 1.8–2.0 times longer than its apical width, middle flagellar segments 1.6–1.7 times longer than wide. Vein 1-R1 0.85–1.00 times as long as pterostigma, 1.1–1.3 times longer than distance from apex of marginal cell to apex of wing. Face width 1.8–1.9 times its height with clypeus. Hypoclypeal depression 1.4–1.5 times wider than shortest distance from depression to eye. Transverse diameter of eye (dorsal view) 2.3–2.6 times longer than temple. Fore wing almost hyaline. 2.0–2.6 mm	***Habrobracon breviradiatus* Tobias, 1957**
–	Antennae thickened; first flagellar segment 1.5–1.8 times longer than its apical width, middle flagellar segments 1.3–1.4 times longer than wide (Fig. 7B). Vein 1-R1 1.3–1.5 times longer than pterostigma, 3.5–5.0 times longer than distance from apex of marginal cell to apex of wing. Face width 1.4–1.6 times its height with clypeus. Hypoclypeal depression 1.1–1.2 times wider than distance from depression to eye. Transverse diameter of eye (dorsal view) 1.5–1.6 times longer than temple. Fore wing faintly darkened in basal half and under pterostigma. 2.0–3.5 mm	***Habrobracon hebetor* (Say, 1836)**
4	Mesosoma usually black, evenly granulose (except ventral side of mesopleuron; Figs 6A, 6C, 6D). Vein 2-SR+M 0.5–0.9 times as long as vein 2-SR, 0.8–1.3 times as long as vein m-cu (Fig. 6B). - Transverse pronotal sulcus deep and often crenulate (Fig. 6D). Lateral area of metanotum usually sculptured. Antero-lateral areas on third metasomal tergite not separated by grooves	**5**
–	Mesosoma reddish-yellow or black, with wide reddish pattern and smoothed areas on mesoscutum, pronotum and lateral side of mesopleuron (Fig. 8E, 8F). Vein 2-SR+M 0.25–0.50 times as long as vein 2-SR, 0.4–0.8 times as long as vein m-cu (Fig. 8B, 9B)	**8**
5	Vein 1-R1 0.8–1.1 times as long as pterostigma complex of species [*Habrobracon didemie* (Beyarslan, 2002), *Habrobracon excisus* Tobias, 1957, *Habrobracon kopetdagi* Tobias, 1957, *Habrobracon marshakovi* (Tobias, 2000), *Habrobracon nigerrimus* Fischer, 1968, *Habrobracon ponticus* (Tobias, 1986), *Habrobracon radialis* Telenga, 1936]
–	Vein 1-R1 1.3–1.5 times longer than pterostigma (Fig. 6B)	**6**
6	Vein 3-SR 0.75–0.95 times as long as vein r (Fig. 6B). OOL 3.0–4.0 times Od; POL 2.3–3.0 times Od (Fig. 6C). Face width 1.7–1.8 times its height with clypeus (Fig. 6E). - Hypoclypeal depression 1.2–1.6 times wider than shortest distance from depression to eye. Middle lobe of mesoscutum without longitudinal stripes of smoothed sculpture (Fig. 6C). Fore wing almost hyaline. Vein 1-R1 2.0–2.5 times longer than distance from apex of marginal cell to apex of wing (Fig. 6B). 2.0–3.2 mm	***Habrobracon concolorans* (Marshall, 1900)**
–	Vein 3-SR 1.4–1.7 times longer than vein r. OOL 2.2–2.8 times Od; POL 1.3–1.9 times Od. Face width 1.4–1.6 times its height with clypeus	**7**
7	Second tergite basally 1.4–1.6 times wider than its median length, coarsely rugose on sides of median convex area. Fore wing almost hyaline. Vein 1-R1 1.8–2.1 times longer than distance from apex of marginal cell to apex of wing. Hypoclypeal depression 1.2–1.3 times wider than minimum distance from depression to eye. Middle lobe of mesoscutum sometimes only with two longitudinal stripes of smoothed sculpture. 2.3–3.2 mm	***Habrobracon crassicornis* (Thomson, 1892)** (*Habrobracon flavosignatus* Tobias, 1957)
–	Second tergite basally 1.7–2.0 times wider than its median length, evenly striate-rugose medially and without median convex area. Fore wing faintly darkened in basal half. Vein 1-R1 3.0–4.5 times longer than distance from apex of marginal cell to apex of wing. Hypoclypeal depression 0.9–1.0 times as wide as shortest distance from depression to eye. Middle lobe of mesoscutum with two smooth longitudinal stripes. 2.5–3.0 mm	***Habrobracon stabilis* (Wesmael, 1838)**
8	Vein 3-SR 1.2–1.3 times longer than vein 2-SR. Vein 1-R1 1.4–1.5 times longer than pterostigma, 5.0–6.0 times longer than distance from apex of marginal cell to apex of wing. Fore wing distinctly darkened in apical half. - Second metasomal tergite coarsely rugose on sides of short, almost smooth and convex median area. Middle lobe of mesoscutum with two smooth longitudinal stripes, but sometimes completely smooth. Median area of first metasomal tergite with roughly crenulate margins. Sculpture of mesosoma and metasoma often smoothed. 2.5–3.0 mm	***Habrobracon lineatellae* Fischer, 1968, stat. resurr.**
–	Vein 3-SR 0.6–1.0 times as long as vein 2-SR (Fig. 8B). Vein 1-R1 1.00–1.35 times as long as pterostigma, 1.7–5.5 times longer than distance from apex of marginal cell to apex of wing. Fore wing hyaline in apical half	**9**
9	Vein 1-R1 1.0–1.2 times as long as pterostigma, 1.7–2.2 times longer than distance from apex of marginal cell to apex of wing. Vein SR1 4.0–4.5 times longer than vein 3-SR. Fore wing almost hyaline. – First flagellar segment 2.1–2.3 times longer than its apical width; middle and penultimate flagellar segments 1.7–1.9 times longer than wide	**10**
–	Vein 1-R1 1.25–1.35 times longer than pterostigma, 2.5–5.5 times longer than distance from apex of marginal cell to apex of wing. Vein SR1 2.4–3.8 times longer than vein 3-SR. Fore wing faintly darkened at least under pterostigma (Figs 8B, 9B). – Side of metanotum smooth (Fig. 8G). Middle lobe of mesoscutum with two smoothed longitudinal stripes (sometimes hardly visible Fig. 8E)	**11**
10	Antennae 23–25-segmented. Vein 3-SR 1.75–1.85 times longer than vein r. Transverse diameter of eye (dorsal view) 1.9–2.2 times longer than temple. Lateral areas of metanotum rugose to areolate with granulation. Propodeum with median keel and rugosity on wide area. OOL 1.4–2.0 times POL; POL 0.9–1.5 times Od. 2.8–3.6 mm	***Habrobracon nygmiae* Telenga, 1936**
–	Antennae 17–19-segmented. Vein 3-SR 1.0–1.4 times as long as vein r. Transverse diameter of eye (dorsal view) 2.6–3.1 times longer than temple. Lateral areas of metanotum faintly granulose to smooth. Propodeum evenly granulose, without median keel. OOL 1.1–1.2 times POL; POL 1.6–2.0 times Od. 2.0–2.6 mm	***Habrobracon telengai* Mulyarskaya, 1955**
11	In female, POL 1.2–1.6 times Od, OOL 1.5–1.7 times POL (male unknown). Metasomal sculpture finer and shiny (same in large specimens; as in Fig. 7F). Lateral and median areas of first tergite almost with same type of sculpture, with carinate furrow (i.e. with carinae going beyond furrow). - In small specimens (body length 1.5–2.0 mm), all tergites weakly sculptured, shagreened; - first tergite with smooth furrow. 1.5–2.7 mm	***Habrobracon viktorovi* Tobias, 1961**
–	In female, POL 1.7–2.2 times Od, OOL 1.2–1.4 times POL (Fig. 8C; in male, 1.3–1.4 and 1.05–1.10 times, respectively). Metasomal sculpture coarser, matt (Fig. 8H, I). Lateral areas of first tergite coarser sculptured in comparison with median area, with areolate furrow (*i.e*. with carinae not going beyond furrow). - In small specimens (body length 2.1–2.2 mm), metasomal sculpture shiny but distinctly visible; - first tergite with smooth furrow. 2.1–2.7 mm	***Habrobracon pillerianae* Fischer, 1980**

## Discussion and conclusions

Our data provide new information about host association and distribution of *Bracon* and *Habrobracon* species. In the literature, there are few reports of *Habrobracon* or *Bracon* species living on *Lobesia botrana* (Table 2). *Habrobracon gelechiae* (Ashmead, 1889), a well-known parasitoid of the potato tuber moth, *Phthorimaea operculella* (Zeller, 1873) (Lepidoptera, Gelechiidae), was introduced into France to control this harmful insect, and has been experimentally reared from larvae of *Lobesia botrana* (Trouvelot 1924). In the vineyards of Sardinia, Delrio et al. (1987) obtained an unidentified *Habrobracon* from the larvae of EGVM, while from the same host in northwest Iran, along with *Habrobracon hebetor*, an unidentified *Bracon* was also obtained (Akbarzadeh Shoukat 2012). As it is well known (Tobias 1961, Shaw and Huddleston 1991, Quicke 2015), *Bracon* and *Habrobracon* are all generalist idiobiont, solitary or gregarious ectoparasitoids, predominantly of Lepidoptera and Coleoptera mature larvae. The data we obtained on *Bracon admotus*, *Habrobracon concolorans*, *Habrobracon hebetor* and *Habrobracon pillerianae* consistently matched with the existing knowledge of these genera.

**Table 2.** List of records of *Habrobracon* and *Bracon* spp. on *Lobesia botrana* arranged in chronological order.

Names as used in scientific publication	Valid names	Geographic area	Authors
*Habrobracon* sp.	*Habrobracon* sp.	Sardinia	Delrio et al. 1987
*Habrobracon* spp.	*Habrobracon* sp.	South Italy	Moleas 1979, 1995; Coscollà 1997
*Habrobracon johannseni* Viereck, 1912	*Habrobracon gelechiae* (Ashmead, 1889)	France, experimentally reared from *Lobesia botrana* larvae	Trouvelot 1924
*Microbracon gelechiae* Ashmead	*Habrobracon gelechiae* (Ashmead)	France	Trouvelot 1924; Thompson 1946; Coscollà 1997
*Habrobracon gelechiae* (Ashmead)	*Habrobracon gelechiae* (Ashmead)	France	Coscollà 1997; Hoffman and Michl 2003
*Bracon* sp.	*Bracon* sp.	Northwest Iran	Akbarzadeh-Shoukat 2012
*Habrobracon* sp.	*Habrobracon* sp.	Northwest Iran	Lotfalizadeh et al. 2012

*Bracon admotus* was described by J. Papp (2000) by examining 14 specimens (13 females and 1 male) from Hungary and Bulgaria. Ten females were obtained by Papp himself, from the larvae of *Byctiscus betulae* (Linnaeus, 1758) (Coleoptera: Attelabidae), which fed on rolled leaves of *Populus tremula* L. Beyarslan and Erdoğan (2012) recorded the species in Turkey.

*Habrobracon concolorans* was re-described by J. Papp in 2008; however, it is still reported as Bracon (Habrobracon) nigricans (Szépligeti) in recent papers (Wang et al. 2012, Zappalà et al. 2012a, b, 2013, Biondi et al. 2013a, b, Beyarslan et al. 2014, Gabarra et al. 2014,). *Habrobracon concolorans* was considered as a synonym of *Bracon stabilis* Wesmael by Belokobylskij et al. (2003) following the World Catalogues by Shenefelt and van Achterberg (1978), and is still considered as such by Beyarslan et al. (2014). This species is a generalist ectoparasitoid of various Lepidopteran families and one coleopteran species of the family Anobiidae. Table 3 reports an updated and revised list of its host species. Biondi et al. (2013b) studied the biology and the developmental strategies of this species on the highly invasive South American tomato leafminer, *Tuta absoluta* (Meyrick) (Lepidoptera Gelechiidae), on tomato in Italy.

**Table 3.** List of the hosts of *Habrobracon concolorans* (Marshall).

Taxa	Main references
**LEPIDOPTERA**	
**GELECHIIDAE**	
*Pexicopia malvella* (Hübner, 1805)	Tobias 1971 1986; Tobias and Belokobilskij 2000; Belokobylskij et al. 2012; Yu et al. 2012
*Phthorimaea operculella* (Zeller, 1873)	Ortu and Floris 1989
*Tuta absoluta* (Meyrick, 1917)	Zappalà et al. 2013; Ghoneim 2014
**NOCTUIDAE**	
*Heliothis maritima* Graslin, 1855	Tobias and Belokobilskij 2000; Belokobilskij et al. 2012
**NYMPHALIDAE**	
*Vanessa cardui* (Linnaeus, 1758)	Tobias and Belokobilskij 2000; Belokobilskij et al. 2012
**PYRALIDAE**	
*Assara terebrella* (Zincken, 1818) (=*Ephestia terebrellum* Zincken nec Zeller)	Györfi 1956; Yu et al. 2012
*Etiella zinckenella* (Treitschke, 1832)	Tobias 1971, 1986; Tobias and Belokobilskij 2000; Belokobylskij et al. 2012; Yu et al. 2012
*Loxostege sticticalis* (Linnaeus, 1761)	Tobias 1971, 1986; Tobias and Belokobilskij 2000; Belokobylskij et al. 2012; Yu et al. 2012
**TORTRICIDAE**	
*Cnephasia sedana* (Constant, 1884)	Tobias 1971, 1986; Tobias and Belokobilskij 2000; Belokobylskij et al. 2012; Yu et al. 2012
*Cydia cosmophorana* (Treitschke, 1835)	Györfi 1956
*Cydia strobilella* (Linnaeus, 1758)	Györfi 1956; Yu et al. 2012
*Lobesia botrana* (Denis & Schiffermüller, 1775)	new host for *Habrobracon concolorans*
**COLEOPTERA**	
**ANOBIIDAE**	
*Ernobius nigrinus* (Sturm, 1837)	Györfi 1956; Yu et al. 2012

*Habrobracon hebetor* has been re-described many times and has a large number of synonyms because of the wide distribution, the broad host range and morphological variability. Regarding its generic attribution, in addition to *Bracon* and *Habrobracon*, it was also once assigned to *Microbracon* Ashmead, 1890 (synonym of *Bracon*). Although they were synonymised for the first time more than 50 years ago (Lal 1942, Puttarudriah and Channa Basavanna 1956, cited through Yu et al. 2012, Tobias 1959), *Habrobracon hebetor* was later separated from *Habrobracon brevicornis* (Wesmael, 1838) on the basis of various morphological characteristics: the number of antennal segments and the length of the antenna, the length of the vein 3-SR of the fore wing in relation to that of the vein r (van Achterberg and Polaszek 1996). Today *Habrobracon brevicornis* is not considered to be valid, given the large variability of the species, and in more recent works, it is reported as a junior synonym of *Habrobracon hebetor* (Papp 2008, Yu et al. 2012). The names *Habrobracon brevicornis* and *Microbracon brevicornis* were also used in the case of *Lobesia botrana* (Table 4), but should be replaced by the name *Habrobracon hebetor*, which is a well-known species: Yu et al. (2012) list 631 papers on it in their database. The host range is also very large. Yu et al. (2012) list 130 species, of which the vast majority are Lepidoptera, but there are also two Coleoptera and one Hymenoptera, Cynipidae. The behavior of *Habrobracon hebetor* is well known and, like the other species of the same genus, it acts as a gregarious larval ectoparasitoid. *Habrobracon hebetor* has been the object of great interest regarding its mass rearing and is used as a biocontrol agent against many pests (Ghimire and Phillips 2014).

**Table 4.** List of records of *Habrobracon hebetor* on *Lobesia botrana* arranged in chronological order.

Names as used in scientific publication	Geographic area	Authors
*Habrobracon* sp.	South Italy	Silvestri 1912; Boselli 1928; Stellwaag 1928
*Habrobracon brevicornis* (Wesmael)	Italy	Silvestri 1912; Goidanich 1934
*Microbracon brevicornis* (Wesmael)	Italy	Silvestri 1912; Thompson 1946; Coscollà 1997
*Habrobracon hebetor* (Wesmael)	South Italy	Moleas 1979
*Bracon* sp. [*Habrobracon* sp.]	Italy	Stellwaag 1928; Hoffman and Michl 2003
*Habrobracon hebetor* (Say)	Greece, laboratory test	Milonas 2005
*Habrobracon hebetor* (Say)	Northwest Iran	Akbarzadeh Shoukat et al. 2008; Akbarzadeh Shoukat 2012; Lotfalizadeh et al. 2012

All these collected Braconinae represent only a minor component of the parasitoid complex we found, mainly represented by the larval endophagous koinobiont *Campoplex capitator* (Hymenoptera: Ichneumonidae). They showed a reduced prevalence, occurring only in three sites out of the nine sampled, while *Campoplex capitator* was found everywhere. These are the typical features of rare taxa, that can play a crucial role in the case of a local and temporal extinction of the main parasitoids (Jain et al. 2014). It is known that generalist parasitoids can play a key role in many insect communities, since they can more easily switch between different species, exerting an influence on the abundance, coexistence and the community structure of many host populations (Jones et al. 2015). In terms of their strong phenotypic plasticity, they are a very important resource as pest-control agents. Their “switching” behavioral skills (Murdoch 1969) make them very suitable to performing rapid changes in the host range, depending on the relative abundance of hosts, or the establishment of a new host in the community. This is a very important behavioural trait under the current climate-change scenario, where increasingly more frequently and intensively, exotic pest introductions occur, often breaking the ecological balance (Stireman et al. 2005, Tylianakis et al. 2008). It is not by chance that two of the first autochthone parasitoids, switched on the introduced exotic pest *Tuta absoluta* (Meyrick) in Europe, are represented by *Habrobracon hebetor* and *Habrobracon concolorans* (Al-Jboory et al. 2012, Doğanlar and Yiğit 2011, Ferracini et al. 2012, Zappalà et al. 2013).

Our findings of three *Habrobracon* and one *Bracon* species living on *Lobesia botrana* larvae in the natural reserve of Migliarino-San Rossore-Massaciuccoli provide important evidence that this wild area could be of great advantage to the surrounding territories. The Tuscan rural landscape is covered in vineyards, where *Lobesia botrana* is the key pest. The vineyard agroecosystem is well integrated with the surrounding areas, rich in natural habitats, and hosts a very diverse braconid fauna (Loni et al. 2012, Belokobylskij et al. 2013, Loni and Lucchi 2014). Indeed, the presence of a natural wild reserve, near to rural and anthropic areas, can play a crucial role as a biodiversity reservoir from which beneficials can spill over and colonize or recolonize perturbed areas.

## Supplementary Material

XML Treatment for Bracon (Glabrobracon) admotus

XML Treatment for Habrobracon concolorans

XML Treatment for Habrobracon hebetor

XML Treatment for Habrobracon pillerianae
